# Serum Galectin-3 as a Potential Predictive Biomarker Is Associated with Poststroke Cognitive Impairment

**DOI:** 10.1155/2021/5827812

**Published:** 2021-12-02

**Authors:** Qian Wang, Kai Wang, Yihong Ma, Simin Li, Yuzhen Xu

**Affiliations:** ^1^Postdoctoral Workstation, Taian City Central Hospital, Taian, Shandong Province, China; ^2^Department of Central Laboratory, Taian City Central Hospital, Shandong First Medical University & Shandong Academy of Medical Sciences, Taian, Shandong Province, China; ^3^State Key Laboratory of Medical Neurobiology-Ministry of Education (MOE) Frontiers Center for Brain Science, Institutes of Brain Science, Fudan University, Shanghai, China; ^4^Department of Neurology, Second Affiliated Hospital of Xuzhou Medical University, Xuzhou, Jiangsu Province, China; ^5^Department of Neurology, Graduate School of Medical Sciences, Kumamoto University, Kumamoto, Japan; ^6^Stomatological Hospital, Southern Medical University, Guangzhou, Guangdong Province, China; ^7^Department of Rehabilitation, The Second Affiliated Hospital of Shandong First Medical University, Taian, Shandong Province, China

## Abstract

**Objective:**

Galectin-3, an inflammatory mediator derived from microglia, participates in the pathophysiological process of various neurological diseases. However, the relationship between galectin-3 and poststroke cognitive impairment (PSCI) remains ambiguous. This research purposed to prove whether serum galectin-3 can predict PSCI.

**Methods:**

In the end, an aggregate of 416 patients with the first acute ischemic stroke (AIS) were continuously and prospectively enrolled in the study. Upon admission, the baseline data of AIS patients were collected, and their serum galectin-3 levels were measured. Three months after the stroke, the Montreal Cognitive Scale (MoCA) was utilized to measure the cognitive function of AIS patients, and PSCI was defined as a MoCA score less than 26 points.

**Results:**

Premised on the MoCA scores, patients were categorized into PSCI cohort and non-PSCI cohort. The two AIS patient cohorts did not exhibit any statistical difference in their baseline characteristics (*p* > 0.05). However, the serum galectin-3 level of AIS patients in the PSCI cohort was considerably elevated (*p* < 0.001). Pearson correlation analysis illustrated that serum galectin-3 level was negatively linked to MoCA score (*r* = −0.396, *p* < 0.05). The findings from the receiver-operating curve (ROC) illustrated that the sensitivity of serum galectin-3 as a possible biomarker for diagnosing PSCI was 66%, and the specificity was 94%. The cut-off value of serum galectin-3 to diagnose PSCI is 6.3 ng/mL (OR = 5.49, *p* < 0.001). Upon controlling for different variables, serum galectin-3 level remained to be an independent predictor of PSCI (*p* < 0.001).

**Conclusions:**

Elevated serum galectin-3 levels are linked to a higher risk of PSCI. Serum galectin-3 could be a prospective biomarker for predicting PSCI.

## 1. Introduction

The World Health Organization (WHO) reports that stroke is ranked as the second greatest contributor of mortality, and the number one contributor of disability in low- and middle-income countries [1]. Cognitive impairment, as a frequent complication of stroke, brings a heavy financial burden to the families of stroke survivors [2]. Research reports have shown that the prevalence of poststroke cognitive impairment (PSCI) is between 20% and 80% and varies with different diagnostic criteria, countries, and races [3]. A recently published Chinese community cross-sectional study reported that the total incidence of PSCI in Chinese is 80.97%, which is significantly higher than that of other regions and ethnicities [4]. At present, there are few effective treatments for stroke, and the pathogenic mechanism of PSCI has also not been fully elucidated. The onset of PSCI may be manifested immediately after stroke, but its symptoms are often delayed. This delay can be seen as a treatment window for PSCI, allowing early intervention to protect cognitive function [5]. Therefore, searching for reliable biomarkers that predict PSCI is particularly important for the prevention as well as treatment of PSCI.

Galectin-3 is a pleiotropic protein that belongs to the lectin family and can be combined with *β*-galactoside [6]. In mammals, the galectin family includes 15 members, and the domain that binds to *β*-galactoside is located in the conserved carbohydrate recognition domain (CRD) [7]. Galectins can be divided into three categories according to the number and function of their CRDs: prototype galectins, tandem-repeat galectins, and chimera-type galectin (galectin-3) [8]. Galectin-3 is a special galectin member, which is the only member containing an n-terminal peptide and a c-terminal CRD. Human galectin-3 is a lectin with a molecular weight of 26 KDa, which can be expressed in various tissues and organs such as the skin, brain, intestinal tract, liver, lungs, heart, and kidney [9]. Galectin-3 is predominantly distributed in the cytoplasm and may also be found on the cell surface, serum, and urine [10]. Galactose-3 has been shown to participate in numerous biological activities, such as cell proliferation, inflammation, fibrosis, host defense, and regulation of apoptosis [11].

In recent years, studies on galectin-3's involvement in various disease processes have been widely reported. However, its research with AIS is relatively small. To date, the association between galectin-3 and PSCI is not yet comprehensively elucidated. The purpose of the current study is to explore whether galectin-3 has the possibility of being a biomarker to predict PSCI. If this correlation is confirmed, it will have important clinical significance for the prevention as well as the treatment of PSCI.

## 2. Methods

### 2.1. Study Population

**Figure 1:**
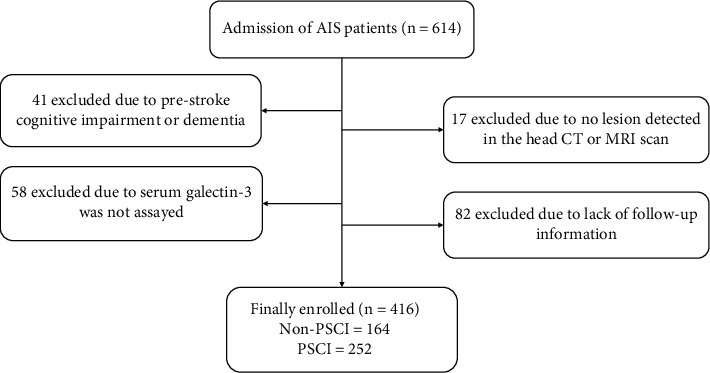
Flow chart of patient selection. AIS: acute ischemic stroke; CT: computerized tomography; MRI: magnetic resonance imaging; PSCI: poststroke cognitive impairment.

The first-onset AIS patients who were treated at the Second Affiliated Hospital of Xuzhou Medical University between January 2018 and December 2020 were selected. The AIS diagnosis is premised on clinical symptoms, brain CT, and MRI images. The AIS diagnosis should also be consistent with the WHO standards [12]. Criteria for patients to be excluded: a past history of PSCI; past history of dementia including Lewy body dementia, Alzheimer's disease, vascular dementia, and other rare forms of dementia; no lesion detected in the head CT or MRI scan; serum galectin-3 was not assayed; lack of follow-up data. In the end, 416 AIS patients were enrolled in the study. The flow chart of patient selection was shown in Figure 1. All patients or family members signed an informed consent form. Approval for this research was provided by the hospital ethics committee, and the research protocol strictly adhered to the requirements of the Declaration of Helsinki.

### 2.2. Baseline Characteristics

Once enrolled, the baseline characteristics of all patients were collected. These baseline characteristics include age, gender, education, BMI, NHISS score, hypertension (HP), diabetes mellitus (DM), hyperlipidemia (HLP), atrial fibrillation (AF), coronary heart disease (CHD), and Trial of ORG 10172 in Acute Stroke Treatment (TOAST) classification. The baseline characteristics of all patients were recorded by professionals for statistical analysis.

### 2.3. Blood Samples Test

Fasting peripheral venous blood was collected from all patients within 24 hours after admission. The venous blood in the serum separation tube (SST) was coagulated at ambient temperature for half an hour and subsequently centrifugated at 1000 × g for 15 min. Participants' sera were used for immediate determination or aliquoted and stored for later use in a refrigerator at a temperature of -20°C. The concentration of serum galectin-3 (Abcam, Cambridge, MA, USA) was detected by enzyme-linked immunosorbent assay (ELISA) based on previous research reports and instructions [13]. Other clinical laboratory tests including leukocyte, hemoglobin, and C-reactive protein (CRP) have also been tested according to ISO 15189 standard.

### 2.4. Neurological Assessment

All patients received NHISS assessment immediately after enrollment, which reflects the degree of neurological damage in patients with AIS. NIHSS consists of 11 projects, each with a score of 0 to 4 for a specific ability. For each item, a score of 0 means that the function of the specific ability is normal, and a higher score indicates that the ability has a certain degree of impairment. The individual scores of each item are summarized to calculate patient's NIHSS total score. The highest score of NHISS can reach 42 points, and the lowest score can reach 0 points, and its score is inversely proportional to nerve damage [14].

Three months after the onset of AIS, all patients underwent the Montreal Cognitive Scale (MoCA) examination to evaluate their cognitive function. The MoCA scale created by Ziad Nasreddine has become a widely used cognitive impairment screening assessment tool since its birth in Montreal in 1996. MoCA includes six subtests: visual space, executive function, naming, attention, abstraction, recall, and positioning, with a total score of 30 points [15]. In this study, a MoCA score of less than 26 was diagnosed as PSCI. AIS patients were categorized into PSCI cohort and non-PSCI cohort according to MoCA score.

All neurological evaluations were completed by experienced neurologists, and they were blind to patient's baseline characteristics.

### 2.5. Statistical Analysis

In this research, continuous variables were articulated as mean ± standard deviation or quartiles, whereas categorical variables were articulated as percentages. The comparison between continuous variables that follow the normal distribution was done utilizing Student's *t*-test, while the comparison between continuous variables with asymmetric distribution was done utilizing the Mann Whitney *U* test. *χ*^2^ test was utilized to examine the differences between categorical variables. According to the quartile of serum galectin-3 levels, the number of cases of PSCI was grouped and calculated. The sensitivity and the specificity of serum galectin-3 levels in the diagnosis of PSCI were evaluated utilizing receiver-operating characteristic (ROC) curve analysis. Calculate the point that maximizes (sensitivity + specificity-1) as the cut-off value. Multivariate logistic regression analysis of the link between serum galectin-3 levels and PSCI. Statistical analyses were conducted utilizing SPSS 23.0 (IBM Corporation, Armonk, NY) software package. 0.05 is used as the threshold to determine whether the difference is significant.

## 3. Results

### 3.1. Baseline Data of Patients according to Cognitive Functions

Finally, our cohort analysis included a total of 416 patients with AIS. We recorded the following baseline characteristics: age, gender, education, BMI, NHISS score, HP, DM, HLP, AF, CHD, TOAST classification, leukocyte, hemoglobin, CRP, and galectin-3. Three months after the onset of AIS, we used the MoCA scale to evaluate the cognitive function of all AIS patients. According to the MoCA score, AIS patients were categorized into 2 cohorts, the PSCI cohort (252 cases) and the non-PSCI cohort (164 cases). We compared the baseline characteristics of the two cohorts, and the results showed no statistical significance (*p* > 0.05). However, the serum galectin-3 levels of AIS patients in the PSCI cohort and non-PSCI cohort were (8.4 ± 2.3) ng/mL and (4.9 ± 1.6) ng/mL, respectively. The level of serum galectin-3 in the PSCI cohort was considerably elevated as opposed to that in the non-PSCI cohort, and the difference was found to be significant (*p* < 0.001).  Table 1 summarizes the baseline features of patients with AIS.

### 3.2. Relationship Serum Galectin-3 Levels and Cognitive Function

Premised on the quartile of serum galectin-3 levels, the prevalence of PSCI is illustrated in Table 2. According to the quartile of serum galectin-3 levels, the prevalence of PSCI is illustrated in Table 2. The results showed a linear relationship between the prevalence of PSCI and the quartile of baseline serum galectin-3 levels in AIS patients: the prevalence of PSCI increased with the increase of serum galectin-3 levels (*p* < 0.001).

We further applied Person correlation analysis to explore the link between serum galectin-3 levels and MoCA scores. The Pearson correlation analysis shows that baseline serum galectin-3 levels are negatively linked to cognitive function 3 months after the onset of stroke (*r* = −0.396, *p* < 0.05).

### 3.3. ROC Analysis of Serum Galectin-3 in the PSCI Diagnosis

**Figure 2:**
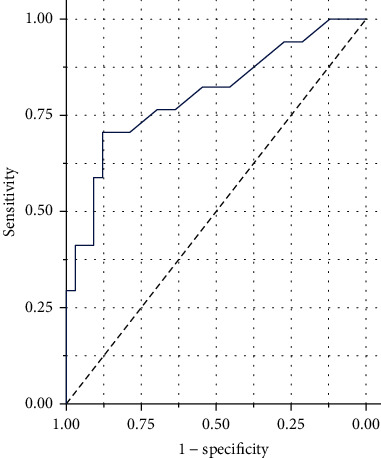
ROC curve for the accuracy of serum galectin-3 levels to diagnose PSCI at 3 months. The AUC of serum galectin-3 levels on PSCI at 3 months was 0.803 with the 95% CI 0.548–0.867. The sensitivity and specificity for serum galectin-3 levels on PSCI at 3 months were 66% and 94%, respectively. Abbreviations: ROC: receiver-operating characteristic; PSCI: poststroke cognitive impairment; AUC: area under the curve; CI: confidence interval.

In order to assess the diagnostic value of serum galectin-3 as a potential marker, ROC curve analysis was used. The area under the curve (AUC) of serum galectin-3 in AIS patients was 0.803, while the diagnostic sensitivity was 66%, and the specificity was 94%. The results of the ROC analysis are shown in Figure 2.

In ROC analysis, we found that the threshold point of serum galectin-3 level for diagnosing PSCI was 6.3 ng/mL. In ROC analysis, we found that the threshold point of serum galectin-3 level for diagnosing PSCI was 6.3 ng/mL. Using this cut-off point, we subsequently analyzed the link between serum galectin-3 levels and PSCI in bivariate analysis.  Table 3 shows the bivariate analysis of serum galectin-3 level classification and PSCI. The results showed that AIS patients with serum galectin-3 levels > 6.3 ng/mL were more likely to develop PSCI than AIS patients with galectin-3 levels below 6.3 ng/mL (OR = 5.49, *p* < 0.001).

### 3.4. Logistic Regression Analysis for the Association of Galectin-3 with PSCI

In order to study whether the serum galectin-3 level can be utilized as a predictor for the diagnosis of PSCI, we performed a multivariate logistic regression analysis. The findings of the logical analysis are illustrated in Table 4. In model 1, after adjustment for gender, age, education, and BMI, a high level of serum galectin-3 is an independent risk indicator for PSCI (OR = 2.34, 95%CI = 2.05‐4.58, *p* < 0.001). In model 2, after further adjustment for vascular risk variables, TOAST classification, and clinical laboratory tests, high levels of serum galectin-3 are also independent risk indicators for PSCI (OR = 1.79, 95%CI = 1.30‐2.11, *p* < 0.001). In model 3, after further adjustment for NHISS, high levels of serum galectin-3 are still an independent risk indicator for PSCI (OR = 1.46, 95%CI = 1.27‐1.83, *p* < 0.001). All results indicate that serum galectin-3 level is an independent risk factor predicting PSCI.

## 4. Discussion

This research examined the difference in serum galectin-3 levels in AIS patients between the PSCI cohort and the non-PSCI cohort. The findings illustrated that the serum galectin-3 level of AIS patients in the PSCI cohort was higher as opposed to that of AIS patients in the non-PSCI cohort. We also found that the incidence of PSCI increased with the increase of galectin-3 levels, suggesting that serum galectin-3 levels are linked to the incidence of PSCI. To evaluate the accuracy of serum galectin-3 levels in the PSCI diagnosis, we conducted ROC analysis and found that serum galectin-3 levels have high sensitivity and specificity for the diagnosis of PSCI. In order to further determine the link between PSCI and serum galectin-3 levels, a variety of models were introduced. We found that serum galectin-3 levels could serve as an independent predictor of PSCI.

A large amount of evidence shows that galectin-3 performs an instrumental function in the normal function of mammalian cells and different pathogenic conditions. Among them, the function of galectin-3 in innate immune inflammation has attracted attention [16]. Galectin-3 is a core modulator of key processes in acute and chronic inflammatory environments [17]. It participates in the process of acute inflammation through opsonization of apoptotic neutrophils, neutrophil clearance, chemoattraction of monocytes/macrophages, and degranulation of mast cells [16]. The process of chronic inflammation is usually accompanied by the formation of fibrosis, and fibroblasts play an important role in this process. Galectin-3 stimulates the expression of a variety of inflammation and chemokines, which can attract monocytes/macrophages to accumulate in the lesion [18]. At the same time, Galectin-3 can bind to integrins on the cell surface to facilitate the bond of neutrophils to vascular endothelial cells [19]. Interestingly, in recent years, it has been discovered that Helicobacter pylori, a common Gram-negative bacillus, whose infection can upregulate the galectin-3 expression in gastric epithelium, damages the blood-brain barrier and causes central system diseases [20]. At the same time, studies have confirmed that galectin-3 can bind to TREM2 on the surface of microglia [21–23], and TREM2 performs an integral function in the selective activation of microglia [24, 25]. In addition, galectin-3 is also believed to be involved in the activation of tobacco amide adenine dinucleotide phosphate (NADPH) and the release of superoxide ions, thereby playing an important role in oxidative stress [26, 27].

Galectin-3 has recently been confirmed to be involved in the processes of a variety of neurological disorders. A Turkish study showed that serum galectin-3 levels are elevated in Parkinson's disease (PD) patients, which might be utilized as a potential noninvasive marker for the diagnosis of PD and advanced stages [28]. A Chinese study showed that the levels of galectin-3 in the brains of patients with Huntington's disease (HD) and mice were higher than those of the control cohort and were related to the severity of the disease. Knockout of galectin-3 in mice has a certain alleviating effect on HD, suggesting that it can be used as a potential therapeutic target for HD [28]. Besides the above neurodegenerative disorders, the function of galectin-3 in neuroimmunity has also been discovered. Jiang et al. found that the lack of galectin-3 can reduce the severity of experimental autoimmune encephalomyelitis [29]. Interestingly, galectin-3 deficiency has similar neuroprotective effects in migraine, acute spinal cord injury (SCI), and traumatic brain injury (TBI) [30–32].

In recent years, galectin 3 has become an emerging biomarker of stroke and cerebrovascular disease. Plasma galectin-3 levels increase in cerebral hemorrhage, which aggravates neuroinflammation and is closely related to poor prognosis, and may become a prognostic biomarker of hemorrhagic stroke [33]. Recent reports indicate that in subarachnoid hemorrhage (SAH), elevated acute plasma galectin-3 levels are linked to post-SAH delayed cerebral ischemia, with no such link with vasospasm, suggesting that galectin-3 could be a prospective novel treatment or research target for brain injury after SAH [34]. The relationship between galectin-3 and ischemic stroke has also been reported in recent years. Wang's team found that high levels of serum galectin-3 can be used as a predictor of poor prognosis for ischemic stroke, independently linked to an enhanced risk of severe disability or death [35]. A recent research report showed that high serum galectin-3 levels can predict the severity of stroke at admittance and the prognosis of stroke at discharge in ischemic stroke patients [36]. Nevertheless, the relationship between galectin-3 and PSCI is still unknown. Up to now, serum galectin-3 levels have been discovered to be increased in Alzheimer's disease (AD), indicating the possibility of its involvement in cognitive impairment [37]. Our current research further expands our understanding of the involvement of galectin-3 in the field of cognitive impairment.

This research has several shortcomings. Firstly, it was based on a cross-sectional study of a small sample, and studies from different regions and ethnic groups are still needed to support our conclusions; secondly, we did not do dynamic monitoring of serum galectin-3 levels and MoCA scores; thirdly, we did not collect the infarct location and volume of AIS, and these data may affect the prognosis; finally, we did not further do interventional experiments in animal and cell models. Nonetheless, our research verified the association between serum galectin-3 levels and PSCI for the first time.

## 5. Conclusions

This study mainly found that serum galectin-3 levels are linked to the cognitive prognosis of AIS. The serum galectin-3 concentration of AIS patients in the PSCI cohort was significantly higher as opposed to that in the non-PSCI cohort. Correlation analysis showed that with the increase of serum galectin-3 levels, the incidence of PSCI in AIS patients gradually increased. Serum galectin-3 level has high accuracy as a biomarker for the diagnosis of PSCI. Logistic regression results indicate that serum galectin-3 levels may be an independent predictor of the onset of PSCI. If the link between serum galectin-3 level and PSCI is additionally confirmed, it may have important value for the ultraearly intervention of PSCI.

## Figures and Tables

**Table 1:** Baseline data of patients according to cognitive functions.

Characteristics	All patients (*n* = 416)	Non-PSCI (*n* = 164)	PSCI (*n* = 252)	*p*
Age, years	64.6 ± 6.9	64.3 ± 7.1	64.8 ± 6.7	0.468
Gender, male, *n* (%)	248 (59.6)	96 (58.5)	153 (60.7)	0.658
Education, years	9.5 ± 1.9	9.6 ± 2.1	9.4 ± 1.8	0.301
BMI, kg/m^2^	24.2 ± 1.6	24.1 ± 1.5	24.3 ± 1.7	0.220
Admission NIHSS	6 (3-8)	6 (3-7)	6 (3-8)	0.712
*Vascular risk factors,n(%)*				
HP	207 (49.8)	80 (48.8)	127 (50.4)	0.747
DM	122 (29.3)	47 (28.7)	75 (29.8)	0.809
HLP	160 (38.5)	61 (37.2)	99 (39.3)	0.668
AF	36 (8.7)	15 (9.1)	21 (8.3)	0.628
CHD	85 (20.4)	33 (20.1)	52 (20.6)	0.849
Current smoking	128 (30.8)	50 (30.5)	78 (31.0)	0.920
Current drinking	149 (35.8)	59 (36.0)	90 (35.7)	0.957
*TOAST classification,n(%)*				
Large-artery atherosclerosis	255 (61.3)	99 (60.4)	156 (62.0)	0.753
Small vessel occlusion	63 (15.1)	24 (14.6)	39 (15.5)	0.815
Cardioembolism	45 (10.8)	19 (11.6)	26 (10.3)	0.684
Other cause	29 (7.0)	12 (7.3)	17 (6.7)	0.823
Undetermined	24 (5.8)	10 (6.1)	14 (5.6)	0.817
*Clinical laboratory tests*				
Leukocyte, 10^9^/L	7.1 ± 2.0	6.9 ± 1.8	7.2 ± 2.1	0.133
Hemoglobin, g/L	128.4 ± 12.1	129.6 ± 13.1	127.7 ± 11.5	0.120
CRP, mg/L	4.2 ± 1.1	4.1 ± 1.0	4.3 ± 1.1	0.061
Galectin-3, ng/mL	7.0 ± 2.0	4.9 ± 1.6	8.4 ± 2.3	<0.001

Abbreviations: PSCI: poststroke cognitive impairment; BMI: body mass index; NHISS: National Institute of Health Stroke Scale; HP: hypertension; DM: diabetes mellitus; HLP: hyperlipidemia; AF: atrial fibrillation; CHD: coronary heart disease; TOAST: Trial of ORG 10172 in Acute Stroke Treatment; CRP: C–reactive protein.

**Table 2:** Relationship between the prevalence of PSCI and serum galectin-3 levels.

Variable	Serum galectin-3 levels				
	Q1 (*n* = 104)	Q2 (*n* = 104)	Q3 (*n* = 104)	Q4 (*n* = 104)	*p* for trend
PSCI, *n* (%)	31 (29.8)	52 (50.0%)	74 (71.2)	95 (91.3)	<0.001

Abbreviations: PSCI: poststroke cognitive impairment.

**Table 3:** Bivariate analysis between PSCI with the categorization of serum galectin-3 levels.

Variables	Non-PSCI		PSCI		OR	95% CI	*p*
	*n*	%	*n*	%			
Galectin-3 levels							
<6.3 ng/mL	116	70.7	77	30.6	Ref		
>6.3 ng/mL	48	29.3	175	69.4	5.49	3.62-5.97	<0.001

Abbreviations: PSCI: poststroke cognitive impairment.

**Table 4:** Logistic regression analysis for the association of galectin-3 with PSCI.

Variables	OR	95% CI	*p*
Model 1	2.34	2.05-4.58	<0.001
Model 2	1.79	1.30-2.11	<0.001
Model 3	1.46	1.27-1.83	<0.001

Model 1 adjusted for age, gender, education, and BMI. Model 2 adjusted for model 1, vascular risk factors, TOAST classification, and clinical biochemical index. Model 3 adjusted for model 2 and NHISS. Abbreviations: PSCI: poststroke cognitive impairment; OR: odds ratio; CI: confidence interval; BMI: body mass index; NHISS: National Institute of Health Stroke Scale.

## Data Availability

The data utilized which corroborated this study's conclusions are accessible once requested from the corresponding author.
